# Astrocyte Bioenergetic Remodeling as a Central Trait of Disrupted Glucocorticoid Signaling: Mechanisms and Implications for Stress Vulnerability

**DOI:** 10.1111/jnc.70394

**Published:** 2026-03-08

**Authors:** Paweł Hanus, Dorota Frydecka, Michał Ślęzak

**Affiliations:** ^1^ Department of Psychiatry Medical University of Wroclaw Wroclaw Poland; ^2^ P4Health, Łukasiewicz Research Network–PORT Polish Center for Technology Development Wroclaw Poland

**Keywords:** astrocyte metabolism, brain energy metabolism, glucocorticoid signaling, neuroenergetics

## Abstract

Glucocorticoids (GCs) are central to the organism's adaptation to stress, coordinating systemic energy distribution and neuroendocrine signaling. While acute effects of GCs are adaptive, chronic GC exposure is increasingly recognized as an important factor contributing to the pathophysiology of neuropsychiatric disorders, such as post‐traumatic stress disorder (PTSD) or major depressive disorder (MDD). A piling evidence points to astrocytes as a central integrator of brain response to stress hormones, including GCs. In this review, we discuss a biphasic regulation of astrocyte metabolism by GCs. According to the hypothesis, astrocytes undergo metabolic adaptations in response to GC: acute exposure leads to the enhancement of astrocyte metabolism through upregulation of glycolysis, mitochondrial activation, and glutamate clearance. In turn, prolonged GC exposure induces a metabolic shift toward branched‐chain amino acid and lipid catabolism, promoting mitochondrial reactive oxygen species (ROS) production and impairing key homeostatic functions, including the astrocyte‐neuron lactate shuttle and calcium signaling. Progressive disruption of astrocytes' supporting function may subsequently lead to synaptic dysregulation and energy imbalance in stress‐related brain pathology. We postulate that a detailed understanding of this dynamic regulation is necessary for targeting astrocyte‐specific metabolic mechanisms in neuropsychiatric disorders.

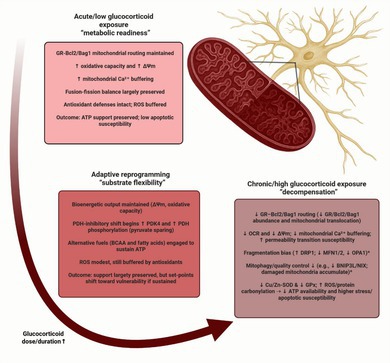

## Graphical abstract

Low/brief glucocorticoid (GC) elevations can enhance mitochondrial output (↑ oxygen consumption rate, OCR; ↑ mitochondrial membrane potential, ΔΨm) and Ca^2+^ buffering, in part via glucocorticoid receptor (GR) trafficking to mitochondria in complexes with Bcl2 and Bag1. With increasing GC dose/duration, an intermediate “substrate flexibility” program may preserve bioenergetic output while shifting metabolism toward reduced pyruvate oxidation (↑ pyruvate dehydrogenase kinase 4, PDK4; ↑ pyruvate dehydrogenase phosphorylation, PDH‐P) and greater reliance on alternative substrates (branched‐chain amino acids, fatty‐acid). Under prolonged/high GC exposure, GR–Bcl2/Bag1 routing is reduced, mitochondrial output and Ca^2+^ buffering decline, redox balance worsens (↑ reactive oxygen species, ROS, ↓ antioxidant enzymes), and mitochondrial quality control is impaired, contributing to reduced ATP availability and increased vulnerability. Abbreviations: ATP, adenosine triphosphate; BCAA, branched‐chain amino acids; Bcl2, B‐cell lymphoma 2; DRP1, dynamin‐related protein 1; GC, glucocorticoid; GPx, glutathione peroxidase; GR, glucocorticoid receptor; MFN1/2, mitofusin‐1/2; OCR, oxygen consumption rate; OPA1, optic atrophy 1; PDH, pyruvate dehydrogenase; PDH‐P, phosphorylated pyruvate dehydrogenase; PDK4, pyruvate dehydrogenase kinase 4; ROS, reactive oxygen species; Cu/Zn‐SOD, copper/zinc superoxide dismutase; BNIP3L/NIX, BCL2 interacting protein 3‐like (NIP3‐like protein X); ΔΨm, mitochondrial membrane potential. Arrows indicate qualitative trends along a dose/duration continuum; thresholds vary by paradigm. *Items marked with an asterisk reflect mechanisms supported strongly in neuronal/whole‐brain systems and are hypothesized to contribute analogously in astrocytes, where direct cell‐type–resolved evidence remains limited.

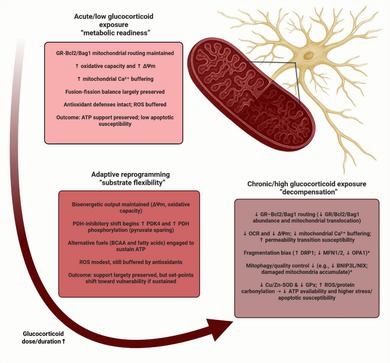

**Abbreviations**
18F‐FDG: 18‐fluorodeoxyglucose
5‐HIAA: 5‐hydroxyindoleacetic acid
ACTH: adrenocorticotropic hormone
Adora2b: adenosine A2B receptor
ANLS: astrocyte–neuron lactate shuttle
AP1: activator protein 1
ATP: adenosine triphosphate
Bag1: Bcl‐2–associated athanogene 1
BBB: blood–brain barrier
BCAA: branched‐chain amino acids
Bcat1: branched‐chain amino acid transaminase 1
BCKA: branched‐chain α‐keto acids
Bckdh: branched‐chain α‐keto acid dehydrogenase (complex)
BNIP3L/NIX: BCL2 interacting protein 3‐like (NIX; mitophagy receptor)
Ca^2+^
: calcium ion
cAMP: cyclic adenosine monophosphate
CORT: corticosterone
CRH: corticotropin‐releasing hormone
DEX: dexamethasone
DRP1: dynamin‐related protein 1
EAAT1: excitatory amino acid transporter 1
EAAT2: excitatory amino acid transporter 2
ER: endoplasmic reticulum
FKBP5: FK506‐binding protein 5
GABA: γ‐aminobutyric acid
GC(s): glucocorticoid(s)
GLAST: glutamate aspartate transporter (EAAT1)
GLT‐1: glutamate transporter 1 (EAAT2)
GLUT1: glucose transporter type 1 (gene Slc2a1)
GLUT3: glucose transporter type 3
GLUT4: glucose transporter type 4 (gene Slc2a4)
GR: glucocorticoid receptor
GRE: glucocorticoid response element
GS: glutamine synthetase
H_2_O_2_
: hydrogen peroxide
HPA (axis): hypothalamic–pituitary–adrenal (axis)
Hsp70: heat shock protein 70
Hsp90: heat shock protein 90
IP3: inositol 1,4,5‐trisphosphate
KO: knockout
MAPK/ERK: mitogen‐activated protein kinase/extracellular signal‐regulated kinase
MDD: major depressive disorder
MFN1/2: mitofusin 1/2
mPTP: mitochondrial permeability transition pore
MR: mineralocorticoid receptor
mRNA: messenger RNA
NF‐κB: nuclear factor kappa B
NLRP3: NLR family pyrin domain–containing 3 (inflammasome)
NMDA: N‐methyl‐D‐aspartate
OXPHOS: oxidative phosphorylation
Pdk4: pyruvate dehydrogenase kinase 4
PET: positron emission tomography
PFC: prefrontal cortex
PI3K: phosphoinositide 3‐kinase
PINK1: PTEN‐induced kinase 1
Ppm1k: protein phosphatase, Mg^2+^/Mn^2+^‐dependent 1K
PTSD: post‐traumatic stress disorder
ROS: reactive oxygen species
Sgk1: serum‐ and glucocorticoid‐regulated kinase 1
siRNA: small interfering RNA
TCA (cycle): tricarboxylic acid (cycle)
TXNIP: thioredoxin‐interacting protein
UTR: untranslated region
ΔΨm: mitochondrial membrane potential

## 1 Introduction

Glucocorticoids (GCs) are key physiological regulators of energy homeostasis in the body. GCs orchestrate cellular adaptation to energetic and psychological stress, with implications ranging from immune‐metabolic balance to neuropsychiatric vulnerability. While systemic effects of GCs' control of metabolism have been extensively studied, the cellular mechanisms mediating GC actions in the brain remain incompletely understood. Deciphering GCs' action in the brain, where energy metabolism is tightly linked to neurotransmitter homeostasis and the function of neural circuits governing behavior is crucial for the progress of therapeutic approaches to psychiatric disorders. Driven by recent transcriptional and metabolic evidence, we focus on discussing metabolic control exerted by GCs on astrocytes: a subtype of glial cells playing crucial roles in neurotransmission, energy metabolism, and homeostasis. Astrocytes are increasingly recognized as a cellular target of GC signaling in the brain and postulated to integrate systemic hormonal signals and local homeostasis. This plasticity initially supports increased neuronal energy demands during stress but becomes maladaptive during chronic GC exposure, leading to mitochondrial dysfunction, impaired gliotransmission, and redox imbalance. We discuss how these processes are temporally regulated and influenced by regional heterogeneity, offering new perspectives on astrocyte contributions to stress vulnerability and treatment of brain pathology associated with stress‐related disorders.

## 2 Glucocorticoid Biology: Synthesis, Function and Mechanisms of Action

### 2.1 GCs Synthesis and Secretion via the HPA Axis

GCs are steroid hormones that, together with sex hormones and mineralocorticoids, are synthesized from cholesterol within the cortex of adrenal glands (Timmermans et al. 2019). Despite the fact that all steroids are structurally highly similar to each other, they differ in terms of their place of origin (GCs are synthesized in the zona fasciculata of the adrenal cortex, whereas mineralocorticoids—in zona glomerulosa) and targets of action. The main route of GCs release is operated by the hypothalamic–pituitary–adrenal (HPA) axis. Stress or metabolic stimuli activate the hypothalamus to secrete corticotropin‐releasing hormone (CRH), which induces adrenocorticotropic hormone (ACTH) release from the anterior pituitary. ACTH acts on the adrenal cortex to stimulate GC synthesis and release. This axis is regulated by negative feedback via circulating GCs acting both on the hypothalamus and pituitary (Timmermans et al. 2019).

### 2.2 GCs Physiological Roles and Systemic Effects

GCs exert physiological impact in a highly tissue‐ and cell‐specific manner (Quatrini and Ugolini 2021; Piechota et al. 2017; Piechota et al. 2025) through two receptors: mineralocorticoid receptor (MR) which shows high affinity to GCs, hence is occupied for most of the time, and glucocorticoid receptor (GR) with much lower affinity than MR, believed to mediate responses to increased levels of GCs, for example, during circadian fluctuations or stress (Timmermans et al. 2019). The GR exists in two main receptor isoforms: GRα, the classical active form, and GRβ, a dominant‐negative isoform that modulates sensitivity to GCs. Variability of GR isoform ratios has been implicated in GCs resistance, inflammation persistence, and steroid treatment refractoriness in chronic diseases (Timmermans et al. 2019; Derijk and de Kloet 2008). Tissue‐specific responses to GCs are a function of the relative expression of the MR and GR and their isoforms, post‐receptor signaling diversity, their specific interactors, cell‐specific molecular context (e.g., local 11β‐hydroxysteroid dehydrogenase activity), and the epigenetic landscape further determining cellular sensitivity to GC exposure (Quatrini and Ugolini 2021; Cooper et al. 2003).

Physiologically, GCs' mediate homeostatic regulation of energy reserves. GCs plasma concentration oscillates in circadian manner, serving as a principal mechanism that couples energy balance and expenditure with organism's activity (So et al. 2009; Kuo et al. 2015). The same system is exploited during stress response. Upon stress exposure, an immediate, fast response is mediated via sympathetic‐adrenomedullary axis (SAM), resulting in the secretion of epinephrine and norepinephrine to the blood, and initiation of the “fight or flight” reaction (McCarty and Gold 1981). Mobilization of SAM activates para‐ventricular nucleus of the hypothalamus, leading to the HPA axis activation an GCs production by adrenal glands (Timmermans et al. 2019; McCarty and Gold 1981). GC‐dependent processes mediate organism adaptation to internal or external stress stimuli. As a result of the negative feedback loop operated by the GR, the hypothalamus and pituitary gland reduce their respective hormone release, maintaining GCs levels within physiological range (Timmermans et al. 2019). This feedback mechanism ensures that GC activity remains tightly regulated under normal conditions.

Fundamental effect of GCs is the increase in glucose pool in the blood via promotion of gluconeogenesis and glycogenolysis in the liver (Timmermans et al. 2019; Tounian et al. 1997) and by restriction of glucose uptake by the main glucose “sink”‐skeletal muscles (Kuo et al. 2015). Along with GCs action, anabolic insulin signaling is being reduced in skeletal muscles (Ohshima et al. 1989) and glucagon secretion is being promoted (Vegiopoulos and Herzig 2007). GCs also act on different energy substrates, increasing more readily available glucose pools via enhancing lipolysis in white adipose tissue (Exton et al. 1972) and promoting lipogenic pathway activity in central fat (Vegiopoulos and Herzig 2007). Each of these actions enables supply of glucose or other energy substrates for organism active phase or stress response.

Other GCs effect, widely used in clinical applications, is immunosuppression by GR‐dependent repression of cyto‐ and chemokines and proinflammatory mediators (Chinenov et al. 2013). Synthetic GCs, like dexamethasone (DEX), exploit these anti‐inflammatory actions but, when overused, can trigger adverse metabolic and neuropsychiatric phenotypes (Dabbah‐Assadi et al. 2022).

### 2.3 GR Control of Transcription

Canonical cellular signaling pathway of GCs consists of genomic and non‐genomic action, which involves interactions with cytosolic, membrane‐bound and mitochondrial GR. Due to their lipophilic nature, GCs diffuse through cell membrane and bind to GR and/or MR (Viho et al. 2019). After diffusion into cells, GCs bind to cytoplasmatic GRs, which form complexes with chaperones (Fkbp5, Hsp90, Hsp70). Upon ligand binding, the complex dissociates allowing GR to translocate into nucleus and bind GC response elements (GREs) in target genes (Surjit et al. 2011). Activated GR can also bind to other transcription factors (NF‐κB, AP1 family members, Stat3) which affects transcriptional activity (Chinenov et al. 2013; Nicolaides et al. 2000) through transactivation, transrepression and other mechanisms (Hudson et al. 2013; Ratman et al. 2013). One of the key regulators of GR transcriptional activity is GR co‐chaperone, Fkbp51. The gene *Fkbp5* is highly responsive to GR activation and Fkbp51 serves as a key mediator of the short‐term negative feedback loop restricting further GC binding and GR nuclear translocation. Elevated Fkbp51 levels hence dampen GR signaling and are associated with GC resistance (Nold et al. 2021; Binder et al. 2004).

### 2.4 Non‐Genomic Pathways and Mitochondrial Regulation

Beside classical genomic action, GCs signaling encompass rapid non‐genomic signaling at the plasma membrane or within the cytoplasm and mitochondria. These fast responses are essential for immediate cellular adaptation and are particularly relevant in tissues with high metabolic turnover, such as the brain. Cytoplasmic actions of GCs are mediated by the activation of intracellular signaling cascades, such as PI3K/Akt, MAPK/ERK or G‐protein coupled pathways, with effects varying by cell type (Panettieri et al. 2019). Downstream effects include the modulation of intracellular Ca^2+^‐either a decrease via adenylyl cyclase–protein kinase A pathway or an increase via phospholipase C, inositol triphosphate (IP_3_) or purinergic 2X receptor activation (Panettieri et al. 2019). Rapid cytoplasmic GR activity may also augment nitrous oxide formation and dependent signaling or reduce N‐methyl‐D‐aspartate (NMDA) elicited currents in neurons (Liu et al. 2007; Di et al. 2009).

Mitochondria convert sugars, lipids, and proteins into ATP via oxidative phosphorylation (OXPHOS), while also regulating metabolic pathways through the tricarboxylic acid (TCA) cycle, maintaining intracellular Ca^2+^ homeostasis, participating in steroidogenesis, and modulating apoptotic signaling. GR can directly bind GREs on mitochondrial genome and regulate OXPHOS through controlling mitochondrial gene expression. The determinants of GR trafficking toward mitochondrial versus nuclear targets remain incompletely recognized, but may involve post‐translational modifications or chaperone‐mediated guidance (Kokkinopoulou and Moutsatsou 2021). Direct modulation of mitochondrial gene expression links GC signaling to energy production and redox balance, making mitochondria not just targets but active integrators of hormonal state. This non‐genomic pathway has found translation to clinical application—for example, in asthma treatment (Alangari 2010), illustrating the importance of understanding tissue– and cell‐specific GC biology for proposing new therapeutic strategies. GC effects on mitochondrial physiology include upregulation of nuclear‐ and mitochondrial‐encoded proteins, activation of OXPHOS complexes, and—when energy demand is high—stimulation of mitochondrial biogenesis (Weber et al. 2002; Rachamim et al. 1995). Recent evidence suggests that GCs rhythms entrain mitochondrial dynamics, aligning bioenergetic activity with diurnal metabolic demands. These rhythmic signals modulate mitochondrial fusion–fission cycles, membrane potential, and oxidative capacity in a time‐of‐day–dependent manner, preparing tissues for periods of high activity or rest. Disruption of this synchrony, for example in shift work or during chronic stress, may destabilize neuronal energetics and contribute to cognitive or mood disturbances (Quattrocelli et al. 2022; Schmitt et al. 2018; Du et al. 2009).

### 2.5 Metabolic Effects of Chronic Exposure to Glucocorticoids

**FIGURE 1:**
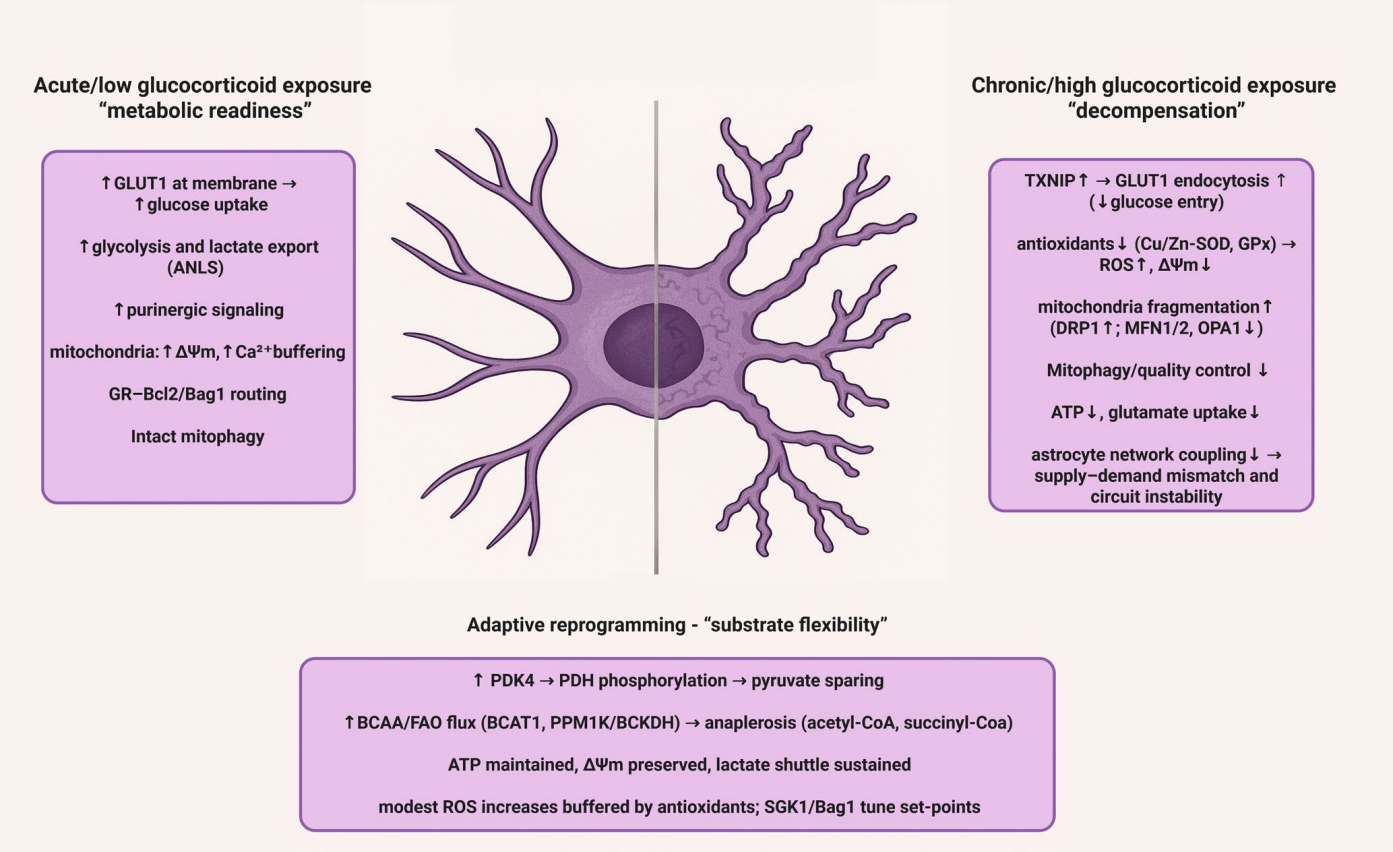
Astrocytic bioenergetic remodeling across glucocorticoid exposure states. Schematic summary of a proposed trajectory from acute/low glucocorticoid exposure (“metabolic readiness”), through adaptive reprogramming (“substrate flexibility”), to chronic/high exposure (“decompensation”). Acute/low exposure is associated with increased glucose uptake (e.g., GLUT1 at the plasma membrane), enhanced glycolysis and lactate export (supporting the astrocyte–neuron lactate shuttle, ANLS), purinergic signaling, and preserved mitochondrial performance (higher mitochondrial membrane potential, ΔΨm, and Ca^2+^ buffering) together with maintained GR–Bcl2/Bag1 mitochondrial routing and mitochondrial quality control. With sustained signaling, an intermediate adaptive state engages a PDK4–PDH inhibitory shift (pyruvate sparing) and increased use of alternative substrates (e.g., branched‐chain amino acids and fatty acids) to replenish the TCA cycle (anaplerosis; acetyl‐CoA, succinyl‐CoA), maintaining ATP output, ΔΨm, and lactate shuttling while shifting redox/signaling set‐points (e.g., SGK1/Bag1). Chronic/high exposure is proposed to promote TXNIP‐dependent limitation of glucose entry (via reduced surface GLUT1), weakened antioxidant capacity (e.g., Cu/Zn‐SOD, GPx), increased ROS with ΔΨm decline, a fragmentation bias (↑DRP1; ↓MFN1/2, ↓OPA1), reduced mitophagy/quality control, impaired ATP availability and glutamate uptake, and reduced astrocytic network coupling—collectively predisposing to supply–demand mismatch and circuit instability. ↑/↓ indicate relative increases/decreases. ANLS, astrocyte–neuron lactate shuttle; ATP, adenosine triphosphate; BCAA, branched‐chain amino acids; BCKDH, branched‐chain α‐ketoacid dehydrogenase complex; Bcl2, B‐cell lymphoma 2; Ca^2+^, calcium; CoA, coenzyme A; Cu/Zn‐SOD, copper/zinc superoxide dismutase; DRP1, dynamin‐related protein 1; FAO, fatty‐acid oxidation; GC, glucocorticoid; GLUT1, glucose transporter 1 (SLC2A1); GPx, glutathione peroxidase; GR, glucocorticoid receptor; MFN1/2, mitofusin‐1/2; OCR, oxygen consumption rate; OPA1, optic atrophy 1; PDK4, pyruvate dehydrogenase kinase 4; PDH, pyruvate dehydrogenase; PINK1, PTEN‐induced kinase 1; PPM1K, protein phosphatase, Mg^2+^/Mn^2+^ dependent 1K; ROS, reactive oxygen species; SGK1, serum‐ and glucocorticoid‐regulated kinase 1; TXNIP, thioredoxin‐interacting protein; ΔΨm, mitochondrial membrane potential.

It is well documented that GCs effects vary depending on the hormone concentration and time of effective exposure, acquiring the biphasic nature (McEwen 2005): adaptive effects of acutely elevated GCs profoundly differ upon sustained exposure which drives allostatic overload and maladaptive changes in energy metabolism (Sterling 2024). This trajectory is summarized in Figure 1. Mechanistically, prolonged GC exposure leads to GR conformational ensembles which specify co‐activator and co‐repressor recruitment and determine patterns of chromatin occupancy (Garside et al. 2004). Concurrent changes in GR trafficking across nuclear, cytosolic, and mitochondrial compartments rebalance classical genomic actions with rapid non‐genomic pathways, producing distinct transcriptional programs and signaling outputs (Timmermans et al. 2019; Panettieri et al. 2019). The systemic outcome of the shift under chronic hypercortisolemia—as in endogenous Cushing's syndrome or during iatrogenic treatment—includes increased hepatic gluconeogenesis and insulin resistance (hyperglycemia), visceral adiposity, muscle proteolysis, and immune dysregulation, yielding clinical features such as fatigue, weight gain, and muscle weakness (Piasecka et al. 2020; Chaudhry and Singh 2024). Likewise, during chronic stress, catecholamines and GCs promote hyperglycemia by increasing hepatic glucose production and limiting peripheral disposal. Catecholamines can also stimulate pancreatic α‐cell glucagon secretion, which further supports hepatic glucose output; however, in insulin‐sufficient conditions hyperglycemia and insulin release suppress glucagon, making glucagon responses variable and context‐dependent across stress states (Gerich et al. 1973; Hamilton et al. 2018). Permanent dysregulation of GC control over systemic metabolism is one of the effects of chronic stress, the main risk factor of psychiatric disorders. Interindividual variation in GC‐regulated gene networks is associated with the risk for psychiatric phenotypes and helps predict antidepressant response, linking GR biology to symptom expression (Menke et al. 2012; Arloth et al. 2015; Carrillo‐Roa et al. 2017; Daskalakis et al. 2024). Consistently, molecular pathways engaging GR appear among the most promising candidates for proposing new therapeutic strategies of stress‐related disorders, including psychiatric disorders (Göver and Slezak 2024).

### 2.6 Brain Vulnerability to Glucocorticoid‐Mediated Metabolic Shifts

In response to elevated plasma GCs target organs shift energy expenditure (Timmermans et al. 2019). The brain, however, is metabolically unique: despite glycogen reserves in astrocytes, it does not possess extensive energy substrate storage, which could be readily available for energetic demands (Öz et al. 2009). This metabolic limitation makes the brain highly sensitive to systemic changes in peripheral glucose availability. Importantly, in the brain, energy metabolism is tightly linked to neurotransmitter homeostasis, decisive for the control of mood and behavior (Yao et al. 2023). Increased circulating glucose levels upon elevated GC secure cerebral energy availability during acute stress or heightened activity, but the reliance on systemic glucose makes the brain particularly vulnerable to GC dysregulation. Chronic GC exposure induces profound alterations in the brain metabolome. For example, targeted metabolomic profiling of rodents exposed to prolonged CORT administration has demonstrated significant reductions in monoamine metabolites, including serotonin, 5‐hydroxyindoleacetic acid (5‐HIAA), acetylcholine, and gamma‐aminobutyric acid (GABA) in mice of mice (Shoji et al. 2024). Importantly, these metabolomic changes were accompanied by behavioral deficits, including reduced locomotor activity, increased anxiety‐ and depression‐like behaviors, and cognitive impairments (Shoji et al. 2024). These effects are similar to clinical manifestations of chronic GCs elevation in humans, which include slowing of information processing and psychomotor speed, reduced attention/working‐memory and executive efficiency, and affective symptoms such as anhedonia, low mood, anxiety, and negative valence bias (Warrington and Bostwick 2006).

Alterations of brain metabolism are also constantly reported across affective disorders. For example, 18F‐FDG PET studies in patients with MDD reported reduced glucose uptake in the anterior cingulate cortex and bilateral insula, alongside increased uptake in the thalamus, hippocampus, posterior cerebellar lobe and the left culmen of the vermis (Su et al. 2014; Fu et al. 2018). Mitochondrial dysfunction, more difficult to assess in human patients, was postulated as causative for neural circuit function based on a translational models (Grandjean et al. 2016; Rosenberg et al. 2023). In turn, neurotransmitter imbalance is a recognized hallmark of psychiatric disorders, with increased evidence pointing to glutamate/glutamine homeostasis as a translational biomarker of depression (Godfrey et al. 2025; Gonsalves et al. 2024). Understanding cellular mechanisms of changes in metabolic pathways induced by chronic stress, largely exerted by GCs, appears crucial for developing better and personalized treatments of psychiatric diseases.

Within the brain, GCs' action is diverse and specific to region or cell type, depending on differences in GR expression, localization, chromatin structure, or context dependent transcriptional regulatory networks (Meijer et al. 2019). Multiple in vitro and in vivo studies have now provided the evidence that transcriptional effects of GCs on glial cells are more robust than in neurons (Piechota et al. 2017; Slezak et al. 2013; Carter et al. 2012; Carter et al. 2013). One example is *Fkbp5*, which showed much higher expression in astrocytes than neurons isolated from the mouse striatum upon systemic DEX administration (Skupio et al. 2015). Interestingly, genetic polymorphisms in *FKBP5* have been linked to increased susceptibility to MDD and PTSD (Binder et al. 2004; Keller et al. 2017; Rao et al. 2016) and found to be one of the most eminent genetic modules linked with those psychiatric diseases in recent, integrative multi‐omics studies (Daskalakis et al. 2024). The presence of variants rendering high risk for MDD led to abrupted *Fkbp5* expression upon GR activation in cultured astrocytes, without noticeable effects in neurons (Nold et al. 2021). These studies suggest that astrocytes may be a key cellular locus of biological processes linking GR signaling in the brain with symptoms of psychiatric disorders.

## 3 Astrocyte Bioenergetics Plasticity Under GC Signaling

### 3.1 Astrocytes as Metabolic and Homeostatic Integrators

Astrocytes are ideally positioned to integrate endocrine signals with the local brain environment: their morphological appearance, with endfeet enwrapping blood vessels in the brain, forming the astrocytic component of the blood–brain barrier (BBB) (Mathiisen et al. 2010) and distal processes ensheathing synapses (Slezak and Pfrieger 2003) makes these cells perfectly suited to bridge synaptic communication with systemic signals, such as hormones. Recent discoveries revealed that astrocytes mediate central effects of elevation of hormones, such as GCs, noradrenaline, insulin, or leptin, leading to reconfiguring neuronal circuitry in a context‐dependent fashion (Murphy‐Royal et al. 2023; Doron et al. 2022; Refaeli et al. 2024).

Astrocytes control brain energy metabolism. Through active control of BBB, they contribute vasoactive signals controlling blood flow and glucose availability (Institoris et al. 2022). They store glycogen reserves which—upon increased energy demands—can be converted to lactate and shuttled to neurons as an energy substrate, f.e. for neuronal activity or maintenance of long term potentiation (Pellerin and Magistretti 1994; Suzuki et al. 2011). Beside shuttling lactate, they also provide neurons with ascorbic acid and glutathione enhancing neuronal antioxidant capacities (Espinoza et al. 2020; Sedlak et al. 2019).

Astrocytes are a subtype of glial cells known to be engaged in homeostatic processes in the central nervous system (PMC 2024). They were shown to sense local synaptic activity in vivo (Slezak et al. 2019) and efficiently deal with its physiological consequences by performing a set of tightly coupled operations. Astrocytes buffer extracellular potassium ions through the functional, connexin‐based syncytium, the process fundamental for maintaining ionic homeostasis and neuronal excitability (Murphy‐Royal et al. 2023). Astrocytes operate the key mechanisms of neurotransmitter turnover, for example, through removing extracellular glutamate and GABA through dedicated transporters and conversion to glutamine, shuttled back to presynaptic neurons for transmitter synthesis (Andersen and Schousboe 2023). These processes are tightly coupled to the energy supply through altering local blood flow and increased glucose metabolism (Doron et al. 2022; Refaeli et al. 2024). Additionally, astrocytes may modulate synaptic functions in many ways, spanning from secretion of synaptogenic factors and mediating synapse elimination (Chung et al. 2015), through release of a variety of neuroactive molecules, ‘gliotransmitters’, such as glutamate, GABA, glycine, ATP, adenosine, endocannabinoids, or serine, each of which is capable of altering properties of synaptic transmission (Araque et al. 2014). This broad biochemical repertoire positions astrocytes not only as passive support cells but as active participants in information processing.

Together, these features underscore astrocytes’ role as “cellular metabolomic hub” that orchestrates both local and systemic homeostasis. Below we summarize the evidence supporting GC‐induced metabolic reprogramming of astrocytes and its consequences for neural circuits. Among functional pathways regulated by GCs in astrocytes are those regulating energy metabolism, redox balance, gliotransmitter cycling and calcium signaling (Piechota et al. 2017; Carter et al. 2012, 2013). Due to the fundamental role of astrocytes in governing brain energy metabolism (Bolaños and Magistretti 2025), in this review we discuss the biphasic nature of GC action in astrocytes: an acute, supportive phase that elevates metabolic readiness and a chronic, maladaptive phase that erodes bioenergetic performance and synaptic support. We note that GC receptors are expressed by virtually all cell types in the brain, and their actions on microglia (Carrillo‐de Sauvage et al. 2013; Frank et al. 2016), neurons (Myers et al. 2014) or oligodendrocytes (Miguel‐Hidalgo 2023) were summarized elsewhere. We acknowledge that neuronal energy demand, microglial inflammatory signaling, and oligodendrocyte myelin formation can co‐determine the metabolic phenotype of astrocytes and remain an important aspect of investigation.

### 3.2 Glucose Metabolism

Glucose entry to the brain is largely insulin‐independent via GLUT1 (expressed in BBB cells, including astroglia) and GLUT3 (neurons) (Koepsell 2020). GC elevations can transiently facilitate neurovascular supply (e.g., through inducing the expression of adrenomedullin (Carter et al. 2012), a vasodilator agent), improving local glucose delivery through enhanced blood flow (Jaszczyk and Juszczak 2021). The role of GR transcriptional regulation of genes engaged in glucose metabolism in astrocytes was shown in vivo: hippocampal astrocytes isolated from transgenic mice with conditional ablation of GR from astrocytes upon systemic DEX (4 h, 100 nM) administration showed blunted increases in the expression of glucose transporter Glut1 (encoded by *Slc2a1*) and pyruvate dehydrogenase kinase 4, *Pdk4* (Tertil et al. 2018). This effect was postulated to be mediated via one of the main GCs effector kinases—Sgk1, known to enhance maximal transport rate of GLUT1 (Palmada et al. 2006). *Sgk1* knockdown in hippocampal astrocyte cultures has not changed level of lactate release and glycogen content upon DEX stimulation, but it blunted glucose uptake, indicating a specific role for Sgk1 in glucose influx under GC influence (Tertil et al. 2018).

GCs profoundly influence astrocytic glucose metabolism. Acute GC exposure was shown to enhance aerobic glycolysis and glycogen breakdown in cultured astrocytes, for example, through to upregulating the expression of glycolytic enzymes aldolase C (AldoC) and *Pdk4* (Carter et al. 2012), resulting in substrate provision to neurons through lactate release (Tanaka et al. 1994; Allaman et al. 2004; Yang et al. 2014). Both, acute stress and exposure to CORT (100 nM, 1 h) has been found to disrupt astrocyte syncytium coupling which coordinates delivery of energy substrates to neurons upon higher energetic demand (Murphy‐Royal et al. 2020). This uncoupling may exemplify the early inflection of the biphasic response at the glial–vascular interface: initial vascular facilitation may be followed by reduced astrocytic gap‐junction coupling and diminished intercellular metabolite transfer, a pattern that intensifies with sustained exposure.

Systemic stress frequently produce hyperglycemia altering brain availability of the main energy substrate. Although elevated levels of circulating GC may normalize within hours to days, stress‐related insulin resistance and catecholamine‐driven hepatic glucose output can sustain elevated glucose and are associated with reduced cerebral glucose uptake (van der Kooij et al. 2018). Rodent studies demonstrated that transport capacity at the BBB can be reduced in hyperglycemic states (Cornford et al. 1995). In astrocytes, GC‐induced TXNIP upregulation decreases surface GLUT1 and glucose uptake (Pan, Pan, et al. 2022; Pan, Zhou, et al. 2022). In turn, insulin‐stimulated GLUT4 translocation operating in selected neuronal populations (e.g., hippocampus) can be impaired by GC exposure (Piroli et al. 2007; Grillo et al. 2009). Hyperglycemia promotes oxidative stress in brain microvascular endothelial cells and is associated with increased BBB permeability, contributing to neurovascular vulnerability (Bogush et al. 2017; Shao et al. 2015). In parallel, ER‐stress signaling has been implicated in diabetes‐related BBB dysfunction while inhibiting ER stress preserved neurovascular barrier integrity in hyperglycemic models (He et al. 2017).

Chronic stress or prolonged GC exposure impairs astrocytic glucose metabolism and astrocyte‐neuron lactate shuttle (ANLS) function, contributing to neuroenergetic decline (as extensively review in (Chamaa et al. 2024)). An early study showed reduced glucose uptake and glycogen concentration in hippocampal astrocyte cultures after 24 h exposure to 100 nM CORT (Tombaugh et al. 1992). Longer, 72 h exposure to 100 nM CORT has been shown to suppress the glycolytic rate, glucose uptake, and GLUT1 expression level, through a mechanisms engaging a molecular glucose sensor—thioredoxin‐interacting protein (TXNIP) (Pan, Zhou, et al. 2022). TXNIP is both glucose‐ and GC‐inducible and acts as an α‐arrestin–like adaptor that binds GLUT1 to promote its clathrin‐mediated endocytosis and/or lysosomal routing, thereby reducing surface GLUT1 and limiting glucose entry (Waldhart et al. 2017; Wu et al. 2013). In parallel, TXNIP binds and inhibits thioredoxin, shifting the cellular redox state toward oxidation; this can elevate ROS signaling and engage redox‐sensitive stress pathways (e.g., NLRP3), further suppressing glycolytic flux and pushing cells toward greater mitochondrial reliance (Zhou et al. 2010; Yoshioka et al. 2012). TXNIP expression level is directly regulated via glucose levels and GCs, forming a negative feedback loop controlling glucose uptake by combining GLUT1 retrieval from the membrane with redox‐driven dampening of glycolysis (Waldhart et al. 2017; Wu et al. 2013). In the study of Pan et al., TXNIP overexpression decreased GLUT1 expression and attenuated depressive‐like behavior, whereas its knockdown restored astrocytic glucose uptake and glycolytic capacity (Pan, Pan, et al. 2022). Notably, this study did not observe a significant lactate increase upon CORT stimulation, consistent with TXNIP‐driven GLUT1 loss uncoupling extracellular glucose availability from astrocytic lactate output and with regional and temporal constraints on lactate dynamics. The TXNIP pathway may also contribute to metabolic shift in astrocytes, where prolonged GC exposure would limits glucose entry after the initial acute GC‐facilitated phase.

Sustained GCs exposure of cultured astrocytes led to upregulation of Pdk4, that is, inhibition of the pyruvate dehydrogenase complex, sparing pyruvate from mitochondrial oxidation (Jha et al. 2012; Connaughton et al. 2010; Kita et al. 2024). This rerouting prioritizes lactate production over oxidative metabolism, reflecting a GC‐driven energy allocation strategy. When pyruvate‐derived carbon entry into the TCA cycle is limited, cells may increase reliance on alternative oxidative substrates. GCs can promote BCAA catabolic programs in peripheral tissues (Shimizu et al. 2011; Huang and Chuang 2000). In the brain, astrocytes exhibit active oxidative BCAA metabolism, with BCAA‐derived carbon entering the TCA cycle via acetyl‐CoA and succinyl‐CoA (Murín et al. 2009; Salcedo et al. 2021) and influx of BCAA may consist up to 40% of total acetyl‐CoA production in cultured astrocytes (Amaral et al. 2011). GCs substantially upregulate expression of *Bcat1* (Branched Chain Amino Acid Transaminase 1) and *Ppm1k* (protein phosphatase, Mg^2+^/Mn^2+^ Dependent 1K) (Piechota et al. 2017; Carter et al. 2012). While Bcat1 transaminates BCAA to branched chained keto acids (BCKA), Ppm1k promotes activation of rate limiting branched chained keto acid dehydrogenase converting BCKA to succinyl‐CoA and acetyl‐CoA which fuel TCA. Taken together, a GC‐driven shift toward BCAA catabolism may provide an anaplerotic bypass to sustain TCA flux (via succinyl‐CoA/acetyl‐CoA) and maintain ATP production when glycolytic pyruvate is limited upon GC exposure. The trade‐off would include greater electron‐transport load and potential ROS elevation, making this rerouting adaptive under sustained demand but liable to become maladaptive with chronic GC exposure. Another possible consequence of that shift may include aberrant neurotransmitter recycling, since in astrocytes the Bcat1–Ppm1k–Bckdh pathway is also engaged in regeneration of glutamate for the glutamate–glutamine cycle, indirectly supporting neuronal transmission (Yudkoff et al. 1994).

### 3.3 Mitochondrial Function

In cortical neuron cultures and rat prefrontal cortex, low‐dose, acute corticosterone increases mitochondrial oxidative capacity, elevates membrane potential (ΔΨm), and enhances mitochondrial Ca^2+^ buffering (Du et al. 2009). By contrast, sustained GC exposure led to decreased oxidative capacity and ΔΨm, and reduced Ca^2+^ buffering (Du et al. 2009). Molecular pathway associated with this plasticity was shown to engage Bcl2‐Bag1 pathway. Upon acute stimulation, GR forms complexes with Bcl2 or Bag1 that translocate to mitochondria, supporting bioenergetic function. Prolonged GC exposure lowered GR, Bcl2, and Bag1 protein abundance and diminished their mitochondrial translocation (Du et al. 2009; Luo et al. 2021). Overexpressing Bag1 was shown to restore GR–Bag1 trafficking deficits, preserving ΔΨm and improving behavior (reduced immobility in the forced swim test and restored sucrose preference) despite high‐dose GC treatment (Luo et al. 2021).

Both, Bcl2 and Bag1 are anti‐apoptotic proteins: Bcl2 blocks mitochondrial outer membrane permeabilization and cytochrome‐c release, while Bag1 stabilizes Bcl2 and coordinates with chaperones to delay its degradation (Nature Reviews Cancer 2024; Aveic et al. 2011). Thus, GC‐dependent mitochondrial routing of GR with Bcl2/Bag1 links bioenergetic support to apoptosis control: enhancing ATP‐generating capacity under low or brief exposure while restraining apoptotic priming, which loss under prolonged exposure couples energetic decline to increased apoptotic susceptibility. This pathway may contribute to astrocyte loss observed in MDD cases (O'Leary and Mechawar 2021).

Another mechanisms of biphasic GC response engages ROS. Hyperglycemia, caused by GCs‐induced increase of pyruvate input to the TCA, enhances electron chain activity and mitochondrial hyperpolarization, thus ROS production (Yu et al. 2006). Acute GC elevations can transiently augment the oxidative capacity while being buffered by intact antioxidant systems. The maladaptive state emerges with chronic GC exposure which suppresses antioxidant defenses (e.g., copper/zinc superoxide dismutase and glutathione peroxidase) and increases ROS generation, promoting protein carbonylation and mitochondrial impairment (Tang et al. 2013; McIntosh et al. 1998; Yu et al. 2011; Madrigal and Madrigal 2001). Stress‐induced hyperglycemia may further increase pyruvate flux into the TCA cycle, increase electron‐transport activity, and elevate mitochondrial membrane potential, thereby amplifying ROS production (Yu et al. 2006). Perturbing GR chaperone regulation also impacts bioenergetics: siRNA knockdown of *Fkbp5* (a negative regulator of GR) in primary mixed cortical cultures reduces oxygen consumption rate and ATP content (Sun et al. 2023). Given that primary glial cultures are ~95% astrocytes, this effect most likely reflects changes in astrocytes bioenergetics. While such cultures are far from the in vivo complexity (Lange et al. 2012; McCarthy and de Vellis 1980) these findings provide mechanistic evidence supporting the influence of GR‐FKBP5 signaling on glial bioenergetics.

The next critical consideration is how chronic exposure disrupts mitochondrial quality‐control mechanisms. In rats, chronic unpredictable mild stress or repeated CORT administration both led to reduced brain mitochondrial function together with decreased expression of fusion proteins (MFN1/2) and antioxidant enzymes, consistent with compromised network maintenance and increased oxidative burden (Liu and Zhou 2012). In vitro, GC receptor activation perturbed mitochondrial morphology and dynamics in neuronal preparations (e.g., dexamethasone‐treated SH‐SY5Y cells) (Suwanjang et al. 2019), while in primary neuronal cultures and rodent brain, acute GC exposure can transiently enhance bioenergetic parameters but prolonged or higher exposure reduces mitochondrial membrane potential (ΔΨm), impairs Ca^2+^ handling, and increases vulnerability to permeability transition and oxidative damage (Du et al. 2009, 2023; Choi et al. 2021). In hippocampal neurons, SH‐SY5Y cells, and mice, GCs also reduced mitophagy flux by downregulating the mitophagy receptor BNIP3L/NIX (reported as PINK1/Parkin‐independent), resulting in mitochondrial accumulation and synaptic impairment (Choi et al. 2021). The evidence for analogical changes in astrocytes remains an important knowledge gap (Choi and Han 2021). Astrocytes rely critically on mitochondrial dynamics: fusion‐dependent remodeling supports perivascular functions after injury (Göbel et al. 2020), while DRP1‐mediated fission controls astrocyte morphogenesis and organization (Rodriguez Salazar et al. 2025). Moreover, astrocytes show age‐related increases in DRP1 and mitochondria fragmentation in cortex (Araujo et al. 2024). This data indicates that chronic GC‐induced alterations of mitochondria dynamics toward fission would probably compromise mitochondrial functions in astrocytes (Rodriguez Salazar et al. 2025).

Interestingly, in midbrain astrocyte cultures, Sgk1 inhibition dampened the H_2_O_2_‐induced proinflammatory NF‐κB signaling and selective inhibition of Sgk1 improved mitochondrial biogenesis, ROS handling capacity and glutamate clearance (Kwon et al. 2021). This data suggest, that while GC‐evoked Sgk1 activation supports metabolic readiness (e.g., facilitates glucose uptake), the sustained GC‐driven Sgk1 activation may contribute to pro‐inflammatory NF‐κB tone, constrained mitochondrial renewal, lowering oxidative resilience and disrupting astrocytic glutamate homeostasis. Mitochondrial mechanisms across exposure states are summarized in graphical abstract.

### 3.4 GC Influence on Astrocytic Calcium Signaling and ATP Release

The biphasic framework extends beyond metabolic substrates. GCs have been shown to modulate Ca^2+^ signaling and ATP release in astrocytes—processes essential for synaptic function and metabolic responsiveness (Madeira et al. 2022; Lu et al. 2022; Koyanagi et al. 2016; Theparambil et al. 2024). Calcium signaling in astrocytes plays a critical role in the modulation of brain function, serving as a fundamental mechanism by which these glial cells communicate both with each other and with neurons, regulate cerebral blood flow, modulate synaptic activity, and maintain homeostasis within the neural environment (Pellerin and Magistretti 1994; Guthrie et al. 1999).

One of the key components regulating Ca^2+^ oscillations is purinergic signaling. When extracellular ATP binds to purinergic receptors on the astrocyte membrane, it triggers the release of Ca2+ from intracellular stores and promotes the Ca^2+^ influx through ion channels (Cotrina et al. 2000; Simard et al. 1999). GCs are known to regulate intracellular Ca^2+^, Ca^2+^−dependent exocytosis of ATP and astrocytic receptors for ATP end metabolite—adenosine (Lu et al. 2022; Koyanagi et al. 2016; Theparambil et al. 2024; Chatterjee and Sikdar 2013). This implicates GC signaling not only in transcriptional control but also in rapid events (Panettieri et al. 2019).

Synthetic GCs, such as methylprednisolone and DEX, were found to elevate astrocyte Ca^2+^ levels in vitro (Simard et al. 1999) A possible mechanism engages GR ability to interact with Ca^2+^ binding protein calreticulin (Lu et al. 2022). Translocation of the GR‐calreticulin complex to the nucleus may reduce cytoplasmic Ca^2+^ −buffering capacity (Lu et al. 2022). Furthermore, GCs potentiated Ca^2+^ response to ATP and bradykinin, which are Ca^2+^ mobilizing agents (Simard et al. 1999), and increased ATP release from astrocyte cultures (Koyanagi et al. 2016). Aggression‐evoked Ca^2+^ signaling was diminished after GR deletion from astrocytes in mice (Koyanagi et al. 2016) and ATP concentrations measured by microdialysis in the medial PFC were significantly decreased in the GR‐KO mice compared to control mice (Koyanagi et al. 2016). GR KO astrocytic cultures also showed reduced ATP levels in the medium after DEX treatment (1 μM, 4 h), indicating GR regulation of astrocytic ATP release (Koyanagi et al. 2016). GC‐dependent ATP release was found to be mediated by lysosomal exocytosis, regulated by the PI3K‐Akt signaling pathway (Koyanagi et al. 2016). This mechanism links transcriptional and vesicular processes in astrocyte responsiveness. Additionally, CORT was shown to enhance release of ATP from spinal astrocytes by Sgk1‐mediated pannexin‐1 hemichannel opening (Koyanagi et al. 2016).

ATP may also have effect on cAMP‐PKA pathway on astrocytes, which is independent of intracellular Ca^2+^ signaling and facilitated by adenosine produced after breakdown of ATP by ectonucleotidase activity (Wu et al. 2023). Response to ATP and adenosine in astrocytes causes enhanced glucose consumption and glycolytic rate, which is accompanied with increased lactate release (Theparambil et al. 2024). Effect has been shown to be mediated by astrocyte specific adenosine receptor Adora2b, for which pharmacological inhibition or genetic knockdown was diminishing cAMP‐PKA astrocyte signaling in response to adenosine and followed lactate release (Theparambil et al. 2024). As release of purines is related to elevated neuronal transmission (Shoji et al. 2024), this mechanism may enable astrocytes to fine‐tune their metabolic output in a manner analogous to ANLS (Suzuki et al. 2011). Adora2b and ectonucleotidase Nt5e expression are upregulated following GC (Carter et al. 2012, 2013). Hence, a purine‐based coupling of energy demand and supply may reinforce GC‐dependent metabolic adaptiveness of astrocytes during stress through increasing astrocyte sensitivity to extracellular purinergic signals and enhancing ATP‐to‐adenosine conversion (Carter et al. 2012, 2013; Wu et al. 2023). This concept is supported by findings of increased lactate shuttling to neurons after GC exposure (Murphy‐Royal et al. 2020; Jha et al. 2012). However, this facilitation appears to be transient: Adora2b expression was found to be decreased in hippocampus and prefrontal cortex after chronic CORT exposure (Carter et al. 2013). The biphasic regulation by GCs may therefore explain reduced lactate shuttle functions in chronic stress or hypercortisolemia.

Elevated intracellular Ca^2+^—potentiated by GCs—may also affect astrocyte mitochondria (Choi and Han 2021; Lu et al. 2022; Koyanagi et al. 2016). As Ca^2+^ signaling is enhanced by GCs, it is plausible that sustained elevations in intracellular Ca^2+^ may promote opening of the permeability of the mitochondrial transition pore (mPTP) (Du et al. 2023; Rizzuto et al. 2009). The mPTP permits small molecules, including protons, to pass through the inner mitochondrial membrane, collapsing the electrochemical gradient, leading to mitochondrial swelling and potential cell death (Du et al. 2023; Javadov and Kuznetsov 2013). This mechanism may contribute to GC‐enhanced Ca^2+^ signaling and mitochondrial vulnerability, particularly in the context of chronic stress.

Furthermore, metabolic profiling revealed pronounced changes in methionine and betaine metabolism, manifested by decreased levels of methionine, choline, dimethylglycine, and adenosine in the brain tissue (Shoji et al. 2024). These metabolites are pivotal for one‐carbon metabolism and methylation capacity, suggesting that chronic stress may alter epigenetic programming via S‐adenosylmethionine–dependent processes. In alignment with previous findings on astrocytic bioenergetics, the observed reduction in adenosine levels may reflect dysregulated ATP breakdown and purinergic signaling, potentially impairing neuron–glia communication and neurovascular coupling. This reinforces prior discussion on GC‐induced modulation of astrocyte Ca^2+^ signaling and gliotransmitter release.

Collectively, these findings demonstrate that GCs potentiate astrocytic Ca^2+^ signaling, ATP release and purinergic sensitivity (Lu et al. 2022; Koyanagi et al. 2016; Theparambil et al. 2024; Wu et al. 2023). While these mechanisms support short‐term neuroenergetic adaptation to stress, they may also exacerbate allostatic overload during prolonged GC exposure, impairing astrocytic and neuronal homeostasis (Bazargani and Attwell 2016).

### 3.5 Glutamate‐Glutamine Shuttle

One specific manifestation of astrocytic adaptation to GCs signaling is the modulation of glutamate‐glutamine shuttle, an essential pathway for synaptic neurotransmitter clearance and metabolic coupling. Removal of excessive glutamate to ensure efficient neurotransmission and prevent glutamate excitotoxicity is one of the main roles of astroglia at glutamatergic synapses. Glutamate is taken up by astrocyte‐specific transporters (EAAT2 and EAAT1), and most of it is converted by glutamine synthetase (GS, encoded by the gene *Glul*). Subsequently, glutamine is recycled back to the neuronal compartment or, in smaller degree, a fraction (reported up to ~20%) is oxidized to α‐ketoglutarate and incorporated to TCA, where its catabolism compensates for the energy expenditure involved in glutamate uptake (McKenna 2013; Todd and Hardingham 2020).

Multiple studies have shown upregulation of *Glul* and *Slc1a2* upon induced GR activation in a dose‐, duration‐ and region‐dependent manner in vivo (Piechota et al. 2017; Carter et al. 2013; Skupio et al. 2015). Notably, the direction of change is not uniform across exposure states: for example, under prolonged/chronic paradigms, *Glul* upregulation may co‐occur with reduced *Slc1a2* in some regions, emphasizing that GC effects on this axis are complex. In primary astrocyte cultures, DEX treatment increased mRNA of *Glul* and GS enzyme activity (Hansson 1989; Jackson et al. 1995) and was shown to induce EAAT2/GLT‐1 (*Slc1a2*) expression (Carter et al. 2012), as well as protein abundance (Zschocke et al. 2005). Notably, GC effects on EAAT2 can also occur post‐transcriptionally: CORT was shown to regulate EAAT2 protein translation via the EAAT2 5′‐UTR, even when EAAT2 mRNA is relatively unchanged, as demonstrated in an astrocyte model system, in neuron–astrocyte mixed cultures, and in vivo (Tian et al. 2007). Because EAAT2/GLT‐1 expression and trafficking are strongly influenced by astrocyte maturation and neuronal activity, the magnitude and direction of GC regulation may depend on culture conditions and developmental stage (Benediktsson et al. 2012).

The upregulation of GS and GLT‐1 upon GC exposure may enhance astrocytic capacity for glutamate clearance and conversion, reinforcing synaptic fidelity and neuroprotection during periods of high neuronal activity or stress‐induced excitation. Indeed, GLT‐1–dependent glutamate uptake was shown to be regulated by GR in primary astrocyte cultures (Zschocke et al. 2005). In turn, decreased expression of GLUL, GLT‐1 (and a second astrocytic glutamate transporter, GLAST) is consistently reported as a deficiency in animal models of chronic stress and in studies of MDD neuropathology (Choudary et al. 2005; Rappeneau et al. 2016; Chen et al. 2014). Together, these findings position glutamate/glutamine homeostasis as a GC‐sensitive node that can shift from compensatory tuning to dysregulation under prolonged stress exposure, potentially contributing to circuit‐level vulnerability in a region‐ and state‐dependent manner.

## 4 Limitations and Future Directions

Despite the recognized role of astrocytes in stress‐related pathologies, the understanding of GC action in these cells remains incomplete. One of the primary limitations of the field lies in the widespread reliance of the current knowledge on in vitro data. These models fail to capture the complete functional profile, spatial heterogeneity, circuit‐specific adaptations, and neuron–glia cross‐talk that are critical to appreciated fine‐tuned responses to GCs. Primary cultures do not reproduce the adult in vivo environment: isolation procedures and serum‐containing media can drive a more reactive phenotype, and baseline expression of mature homeostatic programs (including transporter regulation) depends strongly on the presence of neuronal and vascular cues, (Lee et al. 2017; Prah et al. 2019).

Furthermore, current data are heavily skewed toward transcriptomic endpoints, with a lack of comprehensive, time‐resolved metabolomic profiling. Without detailed information on intracellular fluxes, redox balance, or compartmentalized energy handling, the field cannot yet fully resolve how astrocytes shift between adaptive and maladaptive states under GC pressure. Such studies could help resolve existing inconsistencies in the literature regarding glucose uptake regulation, lactate output, or less studied gliotransmitters, such as glycine or serine, which also play roles in neurotransmission and synaptic plasticity. The time course of GC‐induced metabolic remodeling—including the inflection point of biphasic action of GC, the point in which adaptive states become dysfunctional—remains poorly characterized. This is critical given the proposed biphasic nature of GCs signaling, where the initial increase in metabolic capacity may transition into exhaustion and dysfunction with chronic exposure. Understanding what governs the tipping point from supportive to detrimental could inform about biomarkers for early detection of stress vulnerability and guide intervention strategies aimed at restoring metabolic defects.

Current research has largely overlooked the regulation of non‐glucose‐derived energy substrates in astrocytes under GC influence, particularly branched‐chain amino acid (BCAA) catabolism and fatty acid β‐oxidation. BCAA influx contributes significantly to astrocytic carbon supply (up to 30%) under basal conditions (Amaral et al. 2011). Transcriptomic data suggest that key genes involved in BCAA metabolism—*Bcat1* and *Ppm1k*—are upregulated in response to GCs. Likewise, *Acsl3*, a gene involved in initiating lipid β‐oxidation, also shows increased expression under GC stimulation (Piechota et al. 2017; Carter et al. 2012). The upregulation of these alternative metabolic pathways likely serves to counterbalance the Pdk4‐mediated inhibition of pyruvate entry into the TCA cycle and supports sustained lactate shuttling. Importantly GCs are known to elevate circulating levels of both BCAAs and lipids (Vegiopoulos and Herzig 2007; Kaplan and Shimizu 1963), providing additional substrate for astrocytic metabolism. Nevertheless, excessive reliance on BCAA oxidation and lipid acid β‐oxidation may lead to increased ROS production, especially given the incomplete assembly of mitochondrial complex I in astrocytic supercomplexes, a feature that renders lipid metabolism particularly prone to ROS generation (Rosca et al. 2012; Zhang et al. 2023; Morant‐Ferrando et al. 2023).

While astrocytes appear more resilient than neurons to GC‐induced oxidative stress‐maintaining higher ratios of anti‐apoptotic to pro‐apoptotic proteins (e.g., Bax/Bcl2, Bax/Bcl‐xL) (Yu et al. 2011)–sustained activation of these metabolic routes may contribute to astrocytic mitochondrial stress and allostatic load during chronic GC exposure. This underscores the need to explore the long‐term effects of GC‐induced shifts in astrocyte fuel utilization and redox homeostasis. Understanding what governs the tipping point from supportive to detrimental could inform about biomarkers for early detection of stress vulnerability and guide intervention strategies aimed at restoring metabolic and redox balance before irreversible dysfunction ensues.

The role of sex as a biological variable in astrocytic GC response has been largely neglected, despite compelling evidence of sexually dimorphic stress susceptibility and steroid receptor distribution. For example, the biphasic effect of GCs on mitochondria may be sex‐specific. For example, genetic variants of Fkbp5 render the risk of developing depression upon early life adversity in a sex‐specific manner in humans (Zimmermann et al. 2011; Lavebratt et al. 2010) and mice (Nold et al. 2021). On the metabolic level, male rats exposed to a chronic restraint stress protocol displayed reduced complex II activity in males, while the opposite effect was observed in females. However, stress increased the activity of complexes I‐III in the hippocampus of both sexes, which may enhance mitochondrial ROS production and cumulative oxidative damage (de Souza Mota et al. 2017). The extent to which hormonal milieu, receptor crosstalk or transcriptional plasticity contributes to sex‐specific astrocytic response is an important avenue for further research.

Addressing these questions will require integrated, multi‐level approaches—combining spatial transcriptomics, metabolomic flux profiling, and time‐resolved modeling. Doing so will not only refine our understanding of GC‐astrocyte interaction but also open novel therapeutic frontiers in neuroendocrine and stress‐related neuropathology.

## Author Contributions

**Paweł Hanus:** visualization, conceptualization, writing – original draft, writing – review and editing. **Dorota Frydecka:** supervision, writing – review and editing, funding acquisition. **Michał Ślęzak:** supervision, writing – review and editing, conceptualization, writing – original draft, funding acquisition.

## Funding

This work was supported by Industry PhD Grant (DWD/6/0506/2022) from the Polish Ministry of Science and Higher Education. Project “Astromics” 2023/05/Y/NZ4/00124/WEAVE UNISONO operated by Polish National Science Center.

## Conflicts of Interest

The authors declare no conflicts of interest.

## Data Availability

This review article does not include new datasets generated or analyzed by the authors. All data discussed in this review work is derived from previously published studies, which are cited throughout the manuscript.
